# Monitoring Pharmaceuticals and Personal Care Products in Healthcare Effluent Wastewater Samples and the Effectiveness of Drug Removal in Wastewater Treatment Plants Using the UHPLC-MS/MS Method

**DOI:** 10.3390/molecules29071480

**Published:** 2024-03-27

**Authors:** Lucia Molnarova, Tatana Halesova, Daniela Tomesova, Marta Vaclavikova, Zuzana Bosakova

**Affiliations:** 1 Department of Analytical Chemistry, Faculty of Science, Charles University, Hlavova 8, 128 43 Prague, Czech Republic; lucia.molnarova@natur.cuni.cz; 2 ALS Czech Republic, Na Harfe 223/9, 190 00 Prague, Czech Republic; tatana.halesova@alsglobal.com (T.H.); daniela.tomesova@alsglobal.com (D.T.); marta.vaclavikova@alsglobal.com (M.V.)

**Keywords:** COVID-19, healthcare facilities, wastewater, wastewater treatment plants (WWTPs), pharmaceuticals, personal healthcare products, UHPLC-MS/MS, direct injection

## Abstract

A multi-residue UHPLC–MS/MS analytical method, previously developed for monitoring 52 pharmaceuticals in drinking water, was used to analyse these pharmaceuticals in wastewater originating from healthcare facilities in the Czech Republic. Furthermore, the methodology was expanded to include the evaluation of the effectiveness of drug removal in Czech wastewater treatment plants (WWTPs). Of the 18 wastewater samples analysed by the validated UHPLC-MS/MS, each sample contained at least one quantifiable analyte. This study reveals the prevalence of several different drugs; mean concentrations of 702 μg L^−1^ of iomeprol, 48.8 μg L^−1^ of iopromide, 29.9 μg L^−1^ of gabapentin, 42.0 μg L^−1^ of caffeine and 82.5 μg L^−1^ of paracetamol were present. An analysis of 20 samples from ten WWTPs revealed different removal efficiencies for different analytes. Paracetamol was present in the inflow samples of all ten WWTPs and its removal efficiency was 100%. Analytes such as caffeine, ketoprofen, naproxen or atenolol showed high removal efficiencies exceeding 80%. On the other hand, pharmaceuticals like furosemide, metoprolol, iomeprol, zolpidem and tramadol showed lower removal efficiencies. Four pharmaceuticals exhibited higher concentrations in WWTP effluents than in the influents, resulting in negative removal efficiencies: warfarin at −9.5%, indomethacin at −53%, trimethoprim at −54% and metronidazole at −110%. These comprehensive findings contribute valuable insights to the pharmaceutical landscape of wastewater from healthcare facilities and the varied removal efficiencies of Czech WWTPs, which together with the already published literature, gives a more complete picture of the burden on the aquatic environment.

## 1. Introduction

The pollution of the environment, especially water ecosystems, remains a current topic with a wide impact. Predominant water contaminants, especially pharmaceutical pollutants, pose a significant burden on water quality and aquatic ecosystems and have a potential impact on human health. The global consumption of pharmaceuticals, including non-steroidal anti-inflammatory drugs (NSAIDs), antibiotics and antidepressants, has increased significantly in recent years. This growth is partly attributed to the global COVID-19 pandemic and the overuse of certain groups of drugs [1,2]. The increasing use of pharmaceuticals has led to a corresponding increase in the release of pharmaceuticals and metabolites into wastewater, which contributes to their widespread presence in the environment. Ongoing research is focused on monitoring the occurrence of drugs, their actions and the elimination of their potential toxic effects on the environment [3].

After ingestion, pharmaceutical substances undergo various transformations that lead to the formation of water-soluble metabolites (conjugates) that facilitate their excretion. Medicines excreted in this way end up in wastewater treatment plants (WWTPs) either in their original form or after biotransformation. Substantial amounts of pharmaceutical conjugates, comparable to or exceeding their parent compounds, persist during wastewater treatment in both aqueous and suspended solid phases and subsequently enter directly into receiving waters [4]. Therefore, they are considered emerging contaminants (ECs) due to incomplete regulation regarding environmental health and a lack of understanding of their effects on the environment [5,6,7]. WWTPs are recognized as significant source points of pharmaceutical pollutants, and the initial sources of these aquatic environment pollutants include aquaculture, agriculture, hospitals and pharmaceutical manufacturing plants [8,9,10].

Global studies confirm the presence of pharmaceuticals and their metabolites in aquatic systems. Wastewaters from the Croatian pharmaceutical industry, especially those manufacturers involved in the production of macrolide antibiotics, show different concentrations of antibiotic residues, including sulfonamide antibiotics and their acetylated metabolites [9]. These can affect microbial populations through bacteriostatic and bactericidal effects [11,12,13]. An analysis of wastewater in Spain revealed the presence of 4-aminoantipyrine; beta-blockers, like atenolol; diatrizoic acid; and paracetamol, which were detected in 40% of samples [14]. Similarly, in eastern Spain and northern Italy, the occurrence of commonly used pharmaceuticals such as irbesartan, valsartan, ofloxacin, and sulfamethoxazole in wastewater and surface water has been detected [15]. Furthermore, ibuprofen concentrations of up to 42 μg L^−1^ have been measured in surface waters in Spain [16], while caffeine levels ranging from 36.6 to 59.1 μg L^−1^ have been identified in wastewater samples from the Canary Islands [17]. In addition, a comprehensive multi-residue method applied to untreated wastewater from medical facilities in Barcelona in 2019 detected a variety of pharmaceuticals including ibuprofen, aspirin, levofloxacin and carbamazepine [18]. A study carried out in Rome, Italy, highlighted the persistence of carbamazepine, diclofenac, ibuprofen and gemfibrozil in plant-based WWTPs [19]. In addition, elevated concentrations of paracetamol and paraxanthine and high concentrations of carbamazepine and tramadol metabolites were identified in UK wastewater following treatment at WWTPs [20,21]. Latvia’s main wastewater plant exhibited the highest concentrations of caffeine, paracetamol, ciprofloxacin and ibuprofen [22]. In Norway, a multi-residue method determined the presence of various pharmaceuticals and psychoactive substances in wastewater, underlining the importance of comprehensive monitoring and regulatory measures [23].

Recent studies have shown the adverse effects of pharmaceutical pollutants on aquatic ecosystems by altering the behaviour, physiology and reproduction of living organisms. The contamination of drinking water and the bioaccumulation of pollutants in these organisms can lead to long-term adverse health effects even in human populations. Studies have shown that some pharmaceutical components, even at low concentrations, can interfere with endocrine systems, disrupt hormonal balance and induce the resistance of microbial communities to antibiotics [24,25].

Given the toxic effects of pharmaceutical pollutants and the limited efficiency of WWTPs in eliminating them, there is an urgent need for the development of decontamination technologies for their effective removal. Microbial wastewater treatments have emerged as a valuable solution, utilizing microorganisms as effective agents for decontaminating polluted wastewater [26,27]. In the treatment of pharmaceutical wastewater, physiochemical and biological approaches serve as the primary methods. While the biological treatment method is cost-effective, it may exhibit lower efficiencies for challenging carbon-based wastewater [28]. The electrocoagulation process has demonstrated higher efficiencies in removing persistent pharmaceutically active contaminants [29], whereas conventional biological wastewater treatment methods often lack efficacy in completely eliminating refractory contaminants from pharmaceutical wastewater [30].

Other innovative approaches include advanced oxidation processes (AOPs), the addition of UV radiation, photocatalysis, and the application of ultrasonic waves. Advanced oxidation processes, such as ozone-based treatments and Fenton reactions, demonstrated exceptional efficiency in degrading pharmaceutical compounds by generating highly reactive hydroxyl radicals, resulting in the significant reduction or elimination of contaminants [31]. Similarly, the incorporation of UV radiation into wastewater treatment processes can facilitate the breakdown of pharmaceutical pollutants through photodegradation mechanisms, with high removal rates observed for a wide range of compounds [32]. Photocatalytic techniques, involving the activation of catalysts under light irradiation, offer an environmentally sustainable approach to the degrading of pharmaceutical compounds present in wastewater, achieving substantial removal efficiencies while minimizing energy consumption and chemical usage [33]. Additionally, the application of ultrasonic waves showed efficacy in disrupting pharmaceutical molecules and enhancing their removal from wastewater matrices under controlled conditions [34]. By integrating these advanced methods into wastewater treatment facilities, it is possible to achieve superior removal efficiencies, mitigate the environmental impact of pharmaceutical pollutants, and safeguard water quality for human consumption and ecosystem health.

The European Union has therefore established comprehensive monitoring protocols to assess the concentrations of various substances in water matrices and WWTPs. A list of substances to be monitored throughout the European Union was published by COMMISSION IMPLEMENTING DECISION (EU) 2022/1307 of 22 July 2022 (Directive 2008/105/EC). Among the compounds on this list are pharmaceutical substances, which should be continuously monitored for their occurrence in aquatic ecosystems, and the effectiveness of the removal processes assessed. This approach is intended to inform future regulatory decisions regarding the inclusion of other emerging compounds on the list of substances subject to regular monitoring in aquatic matrices [35].

In this study, the functionality of a previously validated method for determining pharmaceuticals in clean waters (drinking, groundwater, and surface waters) [36] has been verified, specifically for measuring pharmaceuticals in polluted matrices of wastewater. The method of validation included determining the fundamental validation characteristics in wastewater matrices, namely accuracy, precision, linearity, working range, limit of detection, limit of quantification, and selectivity.

The monitoring of wastewater discharged from healthcare facilities in the Czech Republic was conducted and the efficiency of WWTPs was also investigated. Concentrations of pharmaceuticals were measured in the influents and effluents of each WWTP.

## 2. Results

### 2.1. Validation of Method

The UHPLC-MS/MS method was validated for the determination of 52 substances (Table S1) in wastewater. The validation reference matrix was MQ water. Target analytes were quantified using the external standard technique using isotopically labelled drug standards (ISTDs, Table S2) in the validation’s two matrices, wastewater and MQ water. The parameters of the MS/MS detection and final operating parameters of LC-MS/MS method are summarized in Tables S3 and S4, respectively. ISTDs were used in the external standard approach to quantify the target analytes. ISTDs were added to both the fortified matrices and the analysed samples at a level of 0.1 µg L^−1^. Conversion to the recovery of the assigned ISTD (Table S5) was used for the correction of analyte recovery and matrix effects, as stated in [36]. No unwanted interferences were observed in the tested matrices during the validation of the method (Table S3).

The LOQ values range from 1.14 ng mL^−1^ to 251 ng mL^−1^. When examining the LOQ across the entire set of analytes (Table S6), relatively higher limits (>0.05 ng mL^−1^) were observed for certain analytes, such as caffeine, capecitabine, clofibric acid, furosemide, gabapentin, iomeprol, iopromide, naproxen, paclitaxel, and sertraline.

The linear and working range of the method, for wastewater samples, is listed in Table S7. Most of the tested analytes showed a *R*^2^ > 0.999. The exception was the analyte caffeine, which, due to its lower sensitivity and higher limits of detection/quantification, also shows a narrower linear range compared to other analytes.

The precision of the method was evaluated through relative standard deviation (RSD) (%). The RSD for all analytes did not exceed 20% (Tables S8 and S9).

Table S10 summarizes the accuracy of the method, which is expressed as recovery (%). It is evident that the recovery for most analytes is between 70 and 120%. For analytes with a larger linear range, a lower concentration level was prepared. The recovery at the lower spike level is not reported for some analytes (e.g., caffeine, capecitabine, clofibric acid, cyclobenzaprine, diclofenac, fluoxetine, furosemide, gabapentin, gemfibrozil, hydrochlorothiazide, iomeprol, loperamide, naproxen, paclitaxel, salbutamol, sertraline, sotalol, and terbutaline) because the LOQ of these analytes was higher than the tested concentration level. Higher or lower recovery values than recommended for the determination of individual analytes in fortified MQ water blanks and fortified wastewater samples may be caused by matrix effects due to solvent calibration evaluation. Higher values are likely caused by the contamination of the wastewater matrix, which can cause ion enhancement in electrospray ionization (ESI). For caffeine, gabapentin, and iomeprol, the observed values are several times higher. When determining pharmaceuticals in wastewater, the conversion to ISTD is necessary to correct for matrix effects.

### 2.2. Results of Drug Monitoring in Wastewater Samples

**Figure 1:**
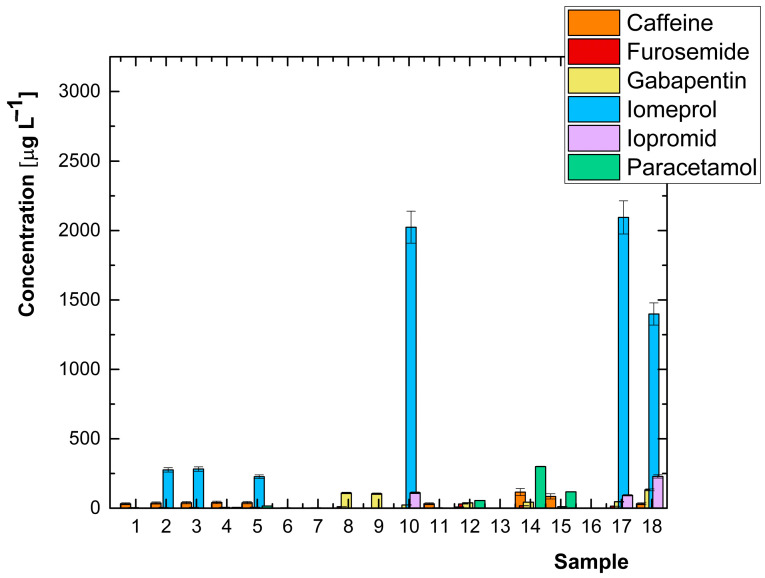
Overview of the most concentrated pharmaceuticals determined in wastewater samples from healthcare facilities in the Czech Republic, *n* = 2.

The validated UHPLC-MS/MS method was used for monitoring pharmaceuticals in wastewater discharged from healthcare facilities in the Czech Republic, which included specialised facilities such as mental health departments or emergency clinics. The results are shown in a graph in Figure 1 and summarised in Table 1. A total of 18 samples were analysed, with at least one analyte determined to be above the limit of quantification in each sample.

Iomeprol, an iodinated contrast medium, was detected in 9 samples at an average concentration level of 702 μg L^−1^, as well as iopromide with an average concentration of 48.8 μg L^−1^. Iodinated contrast agents, commonly used in hospitals for diagnostic purposes, pose challenges due to their highwater solubility and stability.

Gabapentin was determined in all samples with an average concentration of 29.9 μg L^−1^. Caffeine was found in 11 samples with an average concentration of 42.0 μg L^−1^, and furosemide was detected in 8 samples with an average concentration of 9.66 μg L^−1^. Paracetamol was determined in 6 samples with an average concentration of 82.5 μg L^−1^. Other pharmaceuticals with relatively high average concentrations in the samples include metronidazole (7.50 μg L^−1^), sulfamethoxazole (5.92 μg L^−1^), tramadol (3.52 μg L^−1^), trimethoprim (3.06 μg L^−1^), hydrochlorothiazide (2.38 μg L^−1^), diclofenac (1.33 μg L^−1^), naproxen (0.98 μg L^−1^), metoprolol (0.96 μg L^−1^), valsartan (0.91 μg L^−1^), and carbamazepine (0.73 μg L^−1^).

### 2.3. Comparison of the Efficiency of Wastewater Treatment Plants in the Czech Republic

**Figure 2:**
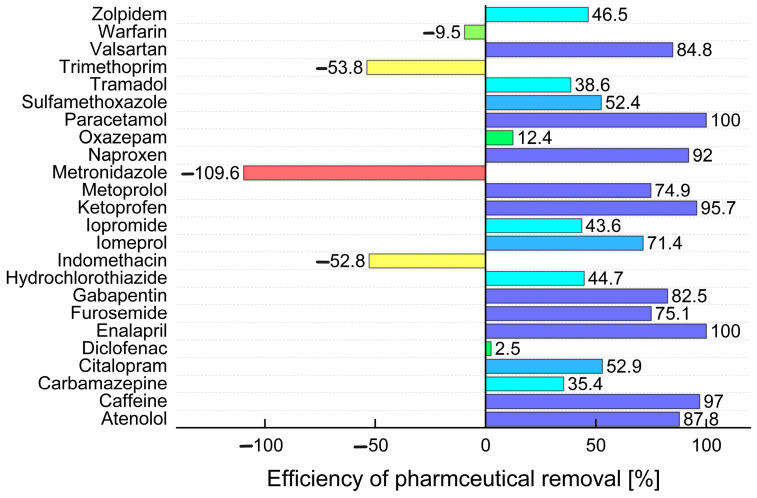
Comparison of the average efficiency of removal of pharmaceuticals in ten WWTPs in the Czech Republic.

The UHPLC-MS/MS method was also used to compare the efficiency of pharmaceutical removal in WWTPs in the Czech Republic. The results are summarized in Table 2 and plotted in graphs in Figure 2. A total of 20 samples from ten WWTPs were analysed, with both influents and effluents from each WWTP being analysed.

Twenty-four compounds were detected in total in at least one raw wastewater sample. Caffeine, diclofenac, furosemide, gabapentin, hydrochlorothiazide, metoprolol, naproxen, tramadol, and valsartan were detected in all raw wastewater samples. It is evident from Figure 2 that the majority of analytes were removed with an efficiency higher than 70% after passing through the WWTPs. Paracetamol was found in the influents of all ten WWTPs with an average concentration of 38.9 μg L^−1^ and was removed with 100% efficiency. Caffeine was also found in all the analysed samples, reaching an average concentration of 82.1 μg L^−1^, and it was removed with a success rate of 97%. Among the analytes removed with an efficiency greater than 80% were: ketoprofen (96%), naproxen (92%), atenolol (88%), valsartan (85%), and gabapentin (83%). A lower removal efficiency was observed for pharmaceuticals such as furosemide (75%), metoprolol (75%), iomeprol (71%), citalopram (53%), sulfamethoxazole (52%), zolpidem (47%), hydrochlorothiazide (45%), iopromide (44%), tramadol (39%), carbamazepine (35%), oxazepam (12%), and diclofenac (3%). Four pharmaceutical analytes showed higher concentrations in the effluents of WWTPs compared to the influents, resulting in negative removal efficiencies: warfarin (−10%), indomethacin (−53%), trimethoprim (−54%), and metronidazole (−110%). A higher concentration of drugs in the effluent compared to the influent may be due to several factors. One possibility is the presence of a matrix suppressing or enhancing the analyte signal during analysis, despite ISs being used, or a potential error in sample preparation or sampling. However, a more likely possibility is the release of metabolised drugs, which may return to their original forms during the wastewater treatment process.

Comparison of the results obtained from the wastewater samples with those from the WWTPs provides good insight into the pathways of pharmaceutical compounds in the treatment system. The analysis of the wastewater samples identified specific pharmaceutical compounds such as iomeprol, iopromide, gabapentin, caffeine, carbamazepine, furosemide, hydrochlorothiazide, metoprolol, naproxen, paracetamol, sulfamethoxazole, tramadol, trimethoprim, and valsartan, indicating their direct entry into the treatment system. On the other hand, the WWTP analysis showed different removal efficiencies for different pharmaceuticals. While compounds such as paracetamol and caffeine were effectively removed with removal rates of 100% and 97%, respectively, others, such as diclofenac and carbamazepine, showed lower removal rates. Interestingly, some drugs, including warfarin and metronidazole, showed negative removal efficiencies, suggesting that their concentrations increased after treatment. Of the drugs detected in both sets of samples, caffeine, carbamazepine, gabapentin, hydrochlorothiazide, iomeprol, iopromide, metoprolol, naproxen, paracetamol, tramadol, trimethoprim, and valsartan were the most common, underscoring their persistence throughout the treatment process. These findings highlight the complex dynamics of drug removal during wastewater treatment and emphasize the necessity for tailored treatment strategies to effectively mitigate drug contamination.

## 3. Discussion

In comparison with studies conducted in Spain, concentrations of paracetamol in the Czech Republic were ten times higher, and additional pharmaceuticals such as atenolol, hydrochlorothiazide, sulfamethoxazole, carbamazepine, and caffeine were also found [14,15]. Approximately the same concentration of caffeine as in the Czech Republic was measured during a study in the Canary Islands [17]. The detected concentration values of paracetamol and naproxen in the Czech Republic were lower than in the United Kingdom [20,21]. In wastewater in Latvia, considerably lower concentrations of caffeine (12 μg L^−1^) and paracetamol (4.2 μg L^−1^) were found than in the Czech Republic [22]. Similar levels of carbamazepine, sulfamethoxazole, carbamazepine, and caffeine were found in wastewater from some healthcare facilities in Canada [37]. The relatively high detected concentrations of analgesics–antipyretics, X-ray contrast media, and bronchodilators in hospital effluent were approximately similar to the values reported in Japan [38]. Even higher concentrations of pharmaceutical active compounds, such as paracetamol (211.93 μg L^−1^), tramadol (76 μg L^−1^), and atenolol (0.43 μg L^−1^) were found in hospital wastewater in Cameroon. On the other hand, the lower analyte concentrations of caffeine (5.8 μg L^−1^), trimethoprim (0.27 μg L^−1^), and sulfamethoxazole 0.16 μg L^−1^) were identified in these waters [39].

In a study conducted in 2014 and 2015 in the Czech Republic, the efficiency of WWTPs was compared, revealing that the highest removal efficiencies were achieved for paracetamol (91%), caffeine (84%), and furosemide (75%), while gabapentin showed the lowest efficiency (14%). Apart from gabapentin, where the validated method showed an 83% removal efficiency, the results align [40]. In a study from 2019 in the Czech Republic and Slovakia, it was found that tramadol, oxazepam, and citalopram were removed in WWTPs with a maximum efficiency of 30%. Compared to this study, higher removal efficiencies were observed for citalopram (53%) and tramadol (39%) [41].

Similar to our monitoring, a maximum removal efficiency of 40% for carbamazepine in WWTPs was determined in a study conducted in the Canary Islands in 2017 [17]. In further studies conducted in Italy in 2014 [19] and in the United Kingdom in 2015 and 2016 [20,21], higher concentrations of pharmaceuticals such as carbamazepine, diclofenac, and tramadol were found after passing through WWTPs.

A Singaporean study investigated the occurrence of 31 emerging contaminants in various water sources, including untreated wastewater, treated wastewater, urban stormwater runoff, agricultural stormwater runoff, and freshwater bodies. Various compounds, including atenolol, caffeine, carbamazepine, indomethacin, ketoprofen, naproxen, and paracetamol exhibited substantial removal in WWTPs with a median removal (MR) rate of 87% or higher. Conversely, certain compounds like carbamazepine, diclofenac, indomethacin, and trimethoprim showed limited removal efficiency in the wastewater treatment processes, with a MR rate of 39% or lower. The removal efficiencies of ECs in this study are comparable with those reported in the literature [6,42].

Significant decreases in the concentrations of acetaminophen (99.8%) and sulfamethoxazole (71%) compounds were confirmed in the WWTPs process, as was the persistence of propranolol and thyroxine compounds. The efficiency of sulfamethoxazole removal in a previous study [4] was higher than in our results (52.4%) but had a lower efficacy, as confirmed by other publications from China [12,13].

Almost complete removal was achieved for acetaminophen, caffeine, and diclofenac in a study conducted in Saudi Arabia in 2023. Atenolol, which is used consistently throughout the year, demonstrated a removal efficiency exceeding 90%. The removal efficiency of various antibiotic pharmaceuticals—cephalexin, ciprofloxacin, ofloxacin, and trimethoprim—exhibited significant variations across the three WWTPs, ranging from 65.29% (ofloxacin) to 99.13% (ciprofloxacin). Moreover, there were no notable differences in their removal efficiency based on distinct treatment processes. However, the removal efficiency of trimethoprim was close to 100%, which does not agree with our findings [43].

The removal efficiencies for numerous antibiotics, antimicrobials, NSAIDs, beta-blockers, anticonvulsants, artificial sweeteners, lipid regulators, and X-ray contrast media can also be negative, such as indomethacin, metronidazole, trimethoprim, and warfarin. This suggests that the concentrations of these ECs in treated effluents were higher than those in the raw influent. The negative removal efficiencies of ECs may be due to various factors. These include the excretion of antibiotics and oral drugs in wastewater samples, the gradual release of pharmaceuticals encapsulated in faecal particles during treatment processes, and potential discrepancies in removal efficiency results when comparing raw influent and treated effluent concentrations [6].

Various factors influence the extent to which these compounds are removed or persist in treatment systems. The solubility of a compound in water, its molecular weight, biological activity, stability, and potential to accumulate in sediments play an important role. Highly soluble compounds, such as diclofenac, tend to remain dissolved, unlike less soluble compounds which are more easily adsorbed and removed. Molecular weight is also important. For example, valsartan is better adsorbed due to its higher molecular weight and achieves removal efficiencies often in excess of 90%. Biological activity determines a compound’s degradability, with atenolol showing moderate biodegradability allowing for effective removal in biological systems, achieving efficiencies over 80%. Chemical stability impacts the compound’s persistence; stable compounds such as iodide contrast mediums (e.g., iopromide) are more complicate to degrade. The ability of compounds to accumulate in sediments, as observed with sulfamethoxazole and tramadol, can be useful in sedimentation processes to enhance removal, with efficiencies up to 60–70% [44,45].

The fluctuation in the occurrence of pharmaceuticals between various WWTPs can be ascribed to several factors. These include divergent patterns of pharmaceutical usage in different locations, variations in WWTP size and capacity, prescription regulations, climate conditions, sampling periods, the population served, and differences in sampling schemes. The primary design focus of most municipal WWTPs is the removal of organic nutrients, including organic carbonaceous, nitrogenous, and phosphorus substances. These facilities may not be specifically engineered to eliminate emerging contaminants, especially persistent and toxic ECs such as antibiotics [6].

Despite WWTPs being major contributors to pharmaceuticals and antibiotic-resistant bacteria (AMRB) in surface waters, the application of ozonation, an advanced water treatment method, is effective in removing a wide range of both pharmaceuticals and AMRB from water samples. These results highlight the importance of reducing pollutant discharge from both WWTPs and medical facilities into surface waters. Nonetheless, X-ray contrast media and other compounds exhibited resistance to ozonation and persisted in water samples even after the treatment [38].

Our findings underscore the necessity for additional research on the environmental fate of chemicals in surface waters and their toxicity to ecosystems. A comprehensive risk assessment of pharmaceuticals in the aquatic environment is essential, as highlighted by previous studies [46,47].

## 4. Materials and Methods

### 4.1. Chemicals and Reagents

A list of analytical standards and isotopically labelled internal standards are included in Tables S1 and S2 in the Supplementary Materials. Acetonitrile and methanol (both of LC-MS grade) were purchased from Honeywell (Honeywell, Charlotte, NC, USA). Acetic and formic acids (both MS grade) were obtained from Sigma-Aldrich^®^ (Sigma-Aldrich^®^, Darmstadt, Germany). Ultrapure water (MQ) was supplied through a Milli-Q water system (Millipore^®^, Billerica, MA, USA).

### 4.2. Sample Collection

Wastewater samples were collected from 18 wastewater outlets originating from healthcare facilities and ten WWTPs in the Czech Republic, where the concentrations of pharmaceutical analytes were monitored in both the influent (inflow) and the effluent (outflow) from the treatment plants. Wastewater samples (1 L) from 18 individual sampling points and ten WWTPs were taken during April and May 2020 in polypropylene (PP) bottles that had been pre-cleaned with deionized water. The bottles were rinsed with deionized water prior to use. Following collection, the grab samples were transported to the laboratory at 4 °C, mixed, homogenized, and stored at −20 °C. The collected samples were immediately placed in a freezer and then analysed without the addition of acidifying or fixing agents. The samples were prepared in duplicate.

### 4.3. Preparation of Samples and Fortified Matrices

The wastewater samples underwent centrifugation at 10,000 RPM for 5 min and were prepared in their undiluted form, and additional dilutions of 10- and 100-fold were also prepared. Samples were then processed following the procedure previously mentioned in [36]. Briefly, the ISTD and analyte stock solutions were added to the water samples at certain concentration levels. As part of the validation, spiking of water samples (10 mL) was performed at two concentration levels, 0.01 ng mL^−1^ and 0.1 ng mL^−1^, by removing the appropriate volume from the stock solution of standards. Samples of the test matrices were prepared in five replicates for each concentration level.

### 4.4. Stock Solutions, Calibration Standards, and Quality Control (QC) Samples, and Preparation of Blank Samples and Laboratory Control Samples (LCS)

The preparation of individual stock solutions, calibration standards, and QC samples is mentioned in the procedure in a previous paper [36]. The blank and laboratory control samples were prepared according to the procedure in [36].

### 4.5. Chromatographic Conditions

A XEVO TQ-S MS analyser (Waters, Milford, MA, USA) paired with an I-class UPLC liquid chromatograph (Waters, USA) was used to conduct the studies. An Acquity UPLC BEH C18 (1.7 μm, 2.1 mm × 100 mm) chromatography column and an Acquity UPLC BEH C18 (1.7 μm) pre-column (Waters, Milford, MA, USA) were used for the chromatographic separations. Gradient elution with 0.01% formic acid in Milli-Q water (A) and 100% methanol (B) was used with the following programme: (min/%B) 0/2, 0.5/2, 5.0/95, 5.1/100, 7.0/100, 7.1/2, and 9.0/2. Both positive and negative modes of electrospray ionization were used to carry out the acquisition. The multiple reaction monitoring (MRM) mode was used for the analyses. For every analyte, two MRM transitions—quantification and qualification—were chosen. Table S3 lists all of the compounds’ MRM transitions, cones and corresponding collision energy, and retention times. The final operational parameters of the UHPLC-MS/MS method are listed in Table S4 and specified in [36].

### 4.6. Method Validation

The characterisation and calculations of the validation parameters are presented in the previous study [36]. The established UHPLC approach has been validated in terms of selectivity, limit of detection and limit of quantification, linear and working range, precision, and accuracy.

## 5. Conclusions

The optimised and validated UHPLC-MS/MS method, with the direct injection of samples, was used for monitoring 52 pharmaceutical analytes in real wastewater samples (a total of 18 samples). At least one analyte was detected above the quantification limit in each sample. Iomeprol was identified in 9 samples at a relatively high concentration (average value of 702 μg L^−1^), as well as iopromide with an average concentration of 48.8 μg L^−1^. Gabapentin was detected in all tested samples, with an average concentration of 29.9 μg L^−1^. Caffeine was found in 11 samples with an average concentration of 42.0 μg L^−1^, and furosemide was present in 8 samples with an average concentration of 9.66 μg L^−1^. Paracetamol was determined in 6 samples with an average concentration of 82.5 μg L^−1^. Other pharmaceuticals identified in samples with relatively high average concentrations include: metronidazole (7.50 μg L^−1^), sulfamethoxazole (5.92 μg L^−1^), tramadol (3.52 μg L^−1^), trimethoprim (3.06 μg L^−1^), hydrochlorothiazide (2.38 μg L^−1^), diclofenac (1.33 μg L^−1^), naproxen (0.98 μg L^−1^), metoprolol (0.96 μg L^−1^), valsartan (0.91 μg L^−1^), and carbamazepine (0.73 μg L^−1^).The results obtained from the wastewater analysis were nearly identical to the values obtained from monitoring studies published in Czech and international scientific studies in recent years.

The UHPLC-MS/MS method was further used to compare the efficiency of pharmaceutical removal in wastewater treatment plants (WWTPs) in the Czech Republic. A total of 20 samples from ten WWTPs were analysed, with both the influent and subsequent effluent from each WWTP being analysed. The majority of analytes were removed with an efficiency higher than 70% after passing through the WWTPs. Paracetamol was found in the influents of all ten WWTPs at an average concentration of 38.9 μg L^−1^ and was removed with 100% efficiency. Caffeine was also found in all analysed samples, with a removal efficiency of 97%. Among the analytes removed with an efficiency greater than 80% are: ketoprofen (96%), naproxen (92%), atenolol (88%), valsartan (85%), and gabapentin (83%). Lower removal efficiency was observed for pharmaceuticals such as: furosemide (75%), metoprolol (75%), iomeprol (71%), citalopram (53%), sulfamethoxazole (52%), zolpidem (47%), hydrochlorothiazide (45%), iopromide (44%), tramadol (39%), carbamazepine (35%), oxazepam (12%), and diclofenac (3%). Four pharmaceutical analytes showed higher concentrations in the effluent of WWTPs compared to the influent, resulting in negative removal efficiencies: warfarin (−10%), indomethacin (−53%), trimethoprim (−54%), and metronidazole (−110%). These findings indicate the importance of reducing the concentrations of environmental pollutants not only at WWTPs but also in healthcare facilities before wastewater is discharged into surface waters and the environment.

As stated in our previous work [36], pharmaceuticals present the same burden on the environment as, e.g., pesticides, plasticisers, or hormones, and thus represent a significant health risk. Therefore, the continuous monitoring of the aquatic environment is necessary, and for this purpose, the developed highly sensitive multi-residue UHPLC-MS/MS method with direct sample injection which significantly simplified the analysis is an advantage.

## Figures and Tables

**Table 1:** Overview of all pharmaceuticals determined in wastewater discharged from 18 healthcare facilities in the Czech Republic (n.d.—not detected).

Analyte	Concentration [μg L^−1^]																	
	1	2	3	4	5	6	7	8	9	10	11	12	13	14	15	16	17	18
Atenolol	n.d.	n.d.	n.d.	n.d.	n.d.	0.301	0.305	n.d.	n.d.	n.d.	n.d.	n.d.	n.d.	n.d.	n.d.	n.d.	n.d.	n.d.
Caffeine	31.7	37.2	39.7	41.2	39.6	n.d.	n.d.	n.d.	n.d.	n.d.	31.5	8.49	n.d.	116.0	84.8	0.597	n.d.	31.5
Carbamazepine	0.742	0.812	0.917	0.846	0.803	0.331	0.333	n.d.	n.d.	n.d.	0.699	n.d.	n.d.	n.d.	n.d.	1.05	n.d.	n.d.
Citalopram	n.d.	n.d.	n.d.	n.d.	n.d.	0.205	0.203	n.d.	n.d.	n.d.	n.d.	n.d.	n.d.	n.d.	0.319	1.04	n.d.	n.d.
Cyclophosphamide	n.d.	n.d.	n.d.	n.d.	n.d.	n.d.	n.d.	n.d.	n.d.	n.d.	n.d.	n.d.	n.d.	n.d.	5.17	n.d.	n.d.	n.d.
Diclofenac	n.d.	n.d.	n.d.	n.d.	n.d.	0.817	0.880	n.d.	n.d.	n.d.	n.d.	n.d.	2.37	n.d.	n.d.	1.27	n.d.	n.d.
Furosemide	n.d.	n.d.	n.d.	n.d.	n.d.	0.378	0.366	10.8	n.d.	n.d.	n.d.	30.5	n.d.	17.7	1.30	1.83	14.4	n.d.
Gabapentin	4.13	5.40	5.77	5.74	6.78	2.58	2.59	109	104	23.2	2.97	38.2	2.28	43.2	1.24	1.56	46.8	132
Hydrochlorothiazide	1.16	2.09	1.69	2.58	2.16	2.39	2.09	n.d.	n.d.	n.d.	0.987	n.d.	n.d.	n.d.	5.91	2.77	n.d.	n.d.
Indomethacin	n.d.	n.d.	n.d.	n.d.	n.d.	0.074	0.065	n.d.	n.d.	n.d.	n.d.	n.d.	n.d.	n.d.	n.d.	n.d.	n.d.	n.d.
Iomeprol	n.d.	276	282	n.d.	227	0.214	0.268	n.d.	n.d.	2024	n.d.	n.d.	n.d.	n.d.	11.4	n.d.	2095	1399
Iopromide	n.d.	1.20	1.28	n.d.	1.20	0.607	0.777	n.d.	n.d.	111	n.d.	n.d.	n.d.	n.d.	1.32	n.d.	92.7	229
Ketoprofen	n.d.	n.d.	n.d.	n.d.	n.d.	n.d.	n.d.	n.d.	n.d.	n.d.	n.d.	n.d.	n.d.	n.d.	15.4	0.090	n.d.	n.d.
Metoprolol	0.632	0.740	0.786	0.902	0.843	1.31	1.30	n.d.	n.d.	n.d.	n.d.	n.d.	n.d.	n.d.	1.46	0.708	n.d.	n.d.
Metronidazole	n.d.	n.d.	n.d.	n.d.	n.d.	n.d.	n.d.	15.7	14.1	n.d.	0.597	n.d.	n.d.	n.d.	n.d.	0.123	6.96	n.d.
Naproxen	1.21	1.22	1.17	1.64	1.25	0.194	0.169	n.d.	n.d.	n.d.	n.d.	n.d.	n.d.	n.d.	n.d.	n.d.	n.d.	n.d.
Oxazepam	n.d.	n.d.	n.d.	n.d.	n.d.	n.d.	0.117	n.d.	n.d.	n.d.	n.d.	n.d.	n.d.	n.d.	n.d.	1.27	n.d.	n.d.
Paracetamol	n.d.	n.d.	0.943	5.42	15.3	n.d.	n.d.	n.d.	n.d.	n.d.	n.d.	55.3	n.d.	300	118	n.d.	n.d.	n.d.
Sertraline	n.d.	n.d.	n.d.	n.d.	n.d.	n.d.	n.d.	n.d.	n.d.	n.d.	n.d.	n.d.	n.d.	n.d.	0.207	n.d.	n.d.	n.d.
Sotalol	n.d.	n.d.	n.d.	n.d.	n.d.	0.055	0.052	n.d.	n.d.	n.d.	n.d.	n.d.	n.d.	n.d.	n.d.	n.d.	n.d.	n.d.
Sulfamethoxazole	n.d.	n.d.	n.d.	n.d.	n.d.	1.14	1.04	6.49	6.62	n.d.	n.d.	20.3	n.d.	7.86	1.15	2.78	n.d.	n.d.
Tramadol	n.d.	n.d.	n.d.	n.d.	n.d.	0.918	0.930	n.d.	n.d.	n.d.	n.d.	7.87	n.d.	6.48	0.291	4.66	n.d.	n.d.
Trimethoprim	0.441	0.759	0.846	0.909	0.877	0.522	0.511	11.4	11.5	n.d.	0.343	5.11	n.d.	4.83	1.40	2.30	3.51	3.71
Valsartan	0.817	0.900	0.697	1.10	0.747	0.242	0.242	n.d.	n.d.	n.d.	0.780	n.d.	n.d.	n.d.	2.81	0.773	n.d.	n.d.
Zolpidem	n.d.	n.d.	n.d.	n.d.	n.d.	n.d.	n.d.	n.d.	n.d.	n.d.	n.d.	n.d.	n.d.	n.d.	n.d.	0.052	n.d.	n.d.

**Table 2:** Overview of the pharmaceuticals determined in the influent (inflow) and the effluent (outflow) from ten WWTPs in the Czech Republic (n.d.—not detected).

Analyte			Concentration [μg L^−1^]																	
	WWTP 1 Inflow	WWTP 1 Outflow	WWTP 2 Inflow	WWTP 2 Outflow	WWTP 3 Inflow	WWTP 3 Outflow	WWTP 4 Inflow	WWTP 4 Outflow	WWTP 5 Inflow	WWTP 5 Outflow	WWTP 6 Inflow	WWTP 6 Outflow	WWTP 7 Inflow	WWTP 7 Outflow	WWTP 8 Inflow	WWTP 8 Outflow	WWTP 9 Inflow	WWTP 9 Outflow	WWTP 10 Inflow	WWTP 10 Outflow
Atenolol	0.093	0.152	n.d.	0.014	0.234	0.214	0.528	0.025	1.87	0.102	3.59	0.088	0.773	0.025	n.d.	n.d.	n.d.	n.d.	0.421	0.300
Caffeine	38.5	2.21	17.1	1.89	21.4	0.959	117.3	1.98	140.7	4.92	160.4	2.09	195.7	1.55	61.6	1.21	45.7	5.90	22.1	2.18
Carbamazepine	0.671	0.849	0.964	0.179	0.139	0.265	0.380	0.432	n.d.	0.003	2.44	0.880	1.42	0.935	0.239	0.336	0.037	n.d.	0.135	0.270
Citalopram	0.206	0.210	0.164	0.150	0.186	0.195	0.825	0.494	4.44	1.44	1.86	0.521	1.35	0.640	0.493	0.711	n.d.	n.d.	0.186	0.222
Diclofenac	3.14	2.03	0.308	0.275	0.213	0.367	3.01	1.79	6.53	3.10	5.21	2.06	5.70	0.496	0.205	0.553	0.050	13.1	0.343	0.322
Enalapril	0.033	n.d.	0.007	n.d.	0.007	n.d.	n.d.	n.d.	0.076	n.d.	n.d.	n.d.	n.d.	n.d.	n.d.	n.d.	n.d.	n.d.	n.d.	n.d.
Furosemide	1.08	1.98	1.71	1.20	1.14	1.19	3.03	1.57	43.2	10.7	2.44	1.16	16.3	1.29	25.8	4.96	2.11	0.009	0.080	0.168
Gabapentin	10.9	12.6	2.00	2.16	4.30	7.33	27.9	3.16	51.7	7.79	19.9	3.92	69.1	3.79	109.9	3.67	2.04	0.372	4.59	8.23
Hydrochlorothiazide	0.635	1.71	0.800	1.27	0.974	1.39	3.96	3.30	11.1	2.52	8.28	3.37	9.03	4.78	9.38	8.76	6.25	0.149	3.04	2.33
Indomethacin	0.104	0.112	0.020	0.046	0.056	0.077	0.049	0.110	0.121	0.103	0.012	0.102	0.164	0.171	0.009	0.090	n.d.	n.d.	0.055	0.088
Iomeprol	0.133	2.90	0.079	n.d.	4.57	1.12	0.110	0.122	222.4	144.0	6.68	2.33	298.1	2.05	n.d.	0.074	n.d.	n.d.	5.33	0.995
Iopromide	n.d.	0.077	n.d.	n.d.	0.052	0.029	0.019	n.d.	n.d.	n.d.	n.d.	0.071	0.804	0.112	n.d.	0.251	n.d.	n.d.	0.083	n.d.
Ketoprofen	0.089	0.045	3.88	n.d.	0.031	0.036	0.012	0.051	1.39	0.070	0.196	0.044	1.26	0.019	n.d.	n.d.	n.d.	n.d.	0.034	0.032
Metoprolol	0.643	0.985	0.700	0.438	0.600	0.800	2.90	1.76	30.8	3.63	12.0	1.82	8.64	2.77	2.40	2.53	3.11	0.026	0.622	0.902
Metronidazole	0.034	0.024	0.006	0.015	0.033	0.112	0.040	0.032	n.d.	0.018	n.d.	0.007	n.d.	0.011	n.d.	n.d.	n.d.	n.d.	0.042	0.118
Naproxen	0.265	0.094	3.49	n.d.	0.270	0.085	4.90	0.651	3.93	0.509	2.49	1.16	12.6	0.078	1.82	n.d.	2.18	n.d.	0.867	0.045
Oxazepam	0.095	0.183	0.008	0.020	0.037	0.077	n.d.	0.012	n.d.	0.020	0.192	0.151	0.216	0.150	n.d.	n.d.	0.202	n.d.	0.040	0.081
Paracetamol	28.7	n.d.	30.3	n.d.	11.0	n.d.	28.6	n.d.	54.5	n.d.	104.2	n.d.	83.7	n.d.	21.4	n.d.	14.7	n.d.	11.8	n.d.
Sulfamethoxazole	0.006	0.009	0.041	0.028	0.503	0.961	1.95	0.678	0.024	0.126	1.82	0.568	4.09	0.557	1.21	0.759	n.d.	n.d.	0.431	1.10
Tramadol	0.231	0.396	0.123	0.247	0.164	0.405	1.97	1.89	17.1	7.11	0.666	1.40	3.59	1.93	n.d.	0.839	0.375	0.022	0.315	0.782
Trimethoprim	0.009	0.106	0.030	0.167	0.152	0.340	0.333	0.316	0.408	0.629	0.206	0.117	0.333	0.160	0.099	0.455	n.d.	n.d.	0.152	0.357
Valsartan	0.347	0.946	0.550	0.038	0.475	0.142	5.45	0.146	29.0	8.11	4.48	0.134	11.3	0.109	8.20	0.145	5.19	0.011	0.477	0.145
Warfarin	0.003	0.006	0.004	0.003	0.003	0.004	0.022	0.007	0.022	0.033	0.024	0.017	0.016	0.019	n.d.	0.016	n.d.	n.d.	0.003	0.003
Zolpidem	0.001	0.002	0.001	0.001	0.001	0.002	0.004	0.005	0.055	0.019	0.005	0.005	0.019	0.006	0.003	0.008	n.d.	n.d.	0.001	0.002

## Data Availability

Data are contained within the article and Supplementary Materials.

## Supplementary Materials

The following supporting information can be downloaded at: https://www.mdpi.com/article/10.3390/molecules29071480/s1. Table S1: List of analytical standards of drugs included in the validation and their characteristics—CAS number, summary formula, relative molecular weight, and structure. Table S2: List of ISTDs included in the validation and their characteristics—ISTD CAS number, summary formula, relative molecular weight, and structure. Table S3: Parameters of the MS/MS detection (MRM transitions of analytes, collision energy, and retention time of analytes) —transitions marked in bold are used as quantification (* designates precursor ions containing two isotopes of chlorine atoms). Table S4: Final operating parameters of the UHPLC-MS/MS method. Table S5: List of ISTDs and assigned analytes in wastewater matrices. Table S6: Overview of values of limits of detection and determination for individual analytes in wastewater. Table S7. Overview of linear range, coefficient of determination (*R*^2^) values, and working range for individual analytes in wastewater. Table S8: Summary of repeatability results expressed as RSD (%). Average of measured concentrations of a given analyte at one concentration level and standard deviation of individual analytes in MQ water. Table S9: Summary of repeatability results expressed as RSD (%), average of measured concentrations of a given analyte at one concentration level and standard deviation of individual analytes in wastewater. Table S10: Method accuracy results expressed as recovery for MQ and wastewater.
