# Supplementary material for: Monitoring Pharmaceuticals and Personal Care Products in Healthcare Effluent Wastewater Samples and the Effectiveness of Drug Removal in Wastewater Treatment Plants Using the UHPLC-MS/MS Method
Source: Molecules. 2024 Mar 27;29(7):1480. doi: 10.3390/molecules29071480 (PMC11013191; doi:10.3390/molecules29071480)
Supplement: Supplementary file 1 [file molecules-29-01480-s001.zip › molecules-2907083-supplementary.pdf]

molecules MDPI

Table S1. List of analytical standards of drugs included in the validation and their characteristics - CAS number, summary formula, relative molecular weight and structure.

	Analyte	CAS	Summary formula (Relative molecular	Structure	Supplier
1	**Anastrozole**	120511-73-1	$C_{17}H_{19}N_5$ (293.4)	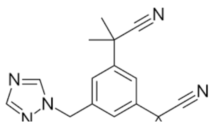	Merck
2	**Atenolol**	29122-68-7	$C_{14}H_{22}N_2O_3$ (266.3)	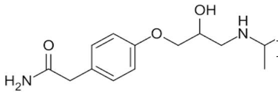	Merck
3	**Azathioprine**	446-86-6	$C_9H_7N_7O_2S$ (277.3)	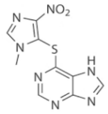	Sigma - Aldrich
4	**Bezafibrate**	41859-67-0	$C_{19}H_{20}ClNO_4$ (361.8)	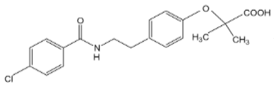	Neochema
5	**Buprenorphine**	52485-79-7	$C_{29}H_{41}NO_4$ (467.6)	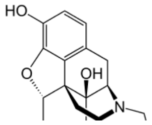	Chromservis
6	**Butorphanol tartrate**	200-659-6	$C_{21}H_{29}NO_2$ (327.5)	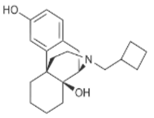	Merck
7	**Caffeine**	58-08-2	$C_8H_{10}N_4O_2$ (194.2)	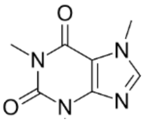	Neochema
8	**Capecitabine**	154361-50-9	$C_{15}H_{22}FN_3O_6$ (359.4)	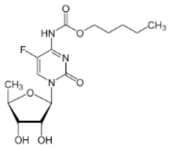	Merck
9	**Carbamazepine**	298-46-4	$C_{15}H_{12}N_2O$ (236.3)	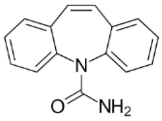	Neochema
10	**Citalopram hydrobromide**	59729-33-8	$C_{20}H_{21}FN_2O \cdot HBr$ (323.1)	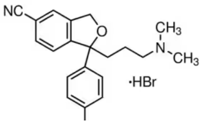	Neochema

	Analyte	CAS	Summary formula (Relative molecular	Structure	Supplier
11	**Clofibric acid**	882-09-7	$C_{10}H_{11}ClO_3$ (214.7)	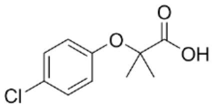	Neochema
12	**Cyclobenzaprine hydrochloride**	6202-23-9	$C_{20}H_{21}N \cdot HCl$ (311.9)	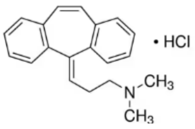	Chromservis
13	**Cyclophosphamide monohydrate**	50-18-0	$C_7H_{15}Cl_2N_2O_2P \cdot H_2O$ (279.1)	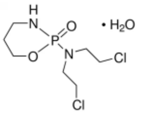	Neochema
14	**Diazepam**	439-14-5	$C_{16}H_{13}ClN_2O$ (284.7)	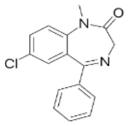	Chromservis
15	**Diclofenac sodium**	15307-79-6	$C_{14}H_{11}Cl_2NO_2$ (296.2)	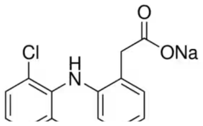	Neochema
16	**Enalapril maleate**	76095-16-4	$C_{20}H_{28}N_2O_5 \cdot C_4H_4O_4$ (492.5)	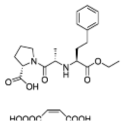	Neochema
17	**Fluoxetine hydrochloride**	59333-67-4	$C_{17}H_{18}F_3NO$ (345.8)	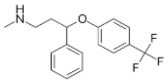	Neochema
18	**Flutamide**	13311-84-7	$C_{11}H_{11}F_3N_2O_3$ (276.2)	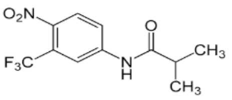	Merck
19	**Furosemide**	54-31-9	$C_{12}H_{11}ClN_2O_5S$ (308.7)	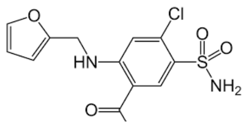	Neochema
20	**Gabapentin**	60142-96-3	$C_9H_{17}NO_2$ (171.2)	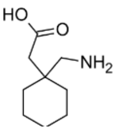	Neochema

	Analyte	CAS	Summary formula (Relative molecular	Structure	Supplier
21	**Gemfibrozil**	25812-30-0	$C_{15}H_{22}O_3$ (250.3)	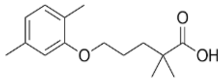	Neochema
22	**Hydrochlorothiazide**	58-93-5	$C_7H_8ClN_3O_4S_2$ (297.7)	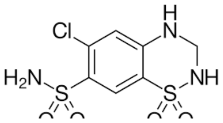	Neochema
23	**Chloramphenicol**	56-75-7	$C_{11}H_{12}Cl_2N_2O_5$ (297.7)	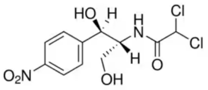	Neochema
24	**Ifosfamide**	3778-73-2	$C_7H_{15}Cl_2N_2O_2P$ (261.1)	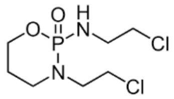	Neochema
25	**Indomethacin**	53-86-1	$C_{19}H_{16}ClNO_4$ (357.8)	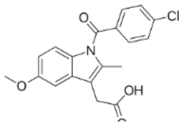	Neochema
26	**Iomeprol**	78649-41-9	$C_{17}H_{22}I_3N_3O_8$ (777.1)	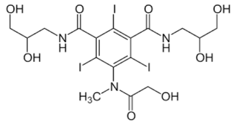	Neochema
27	**Iopamidol**	60166-93-0	$C_{17}H_{22}I_3N_3O_8$ (777.1)	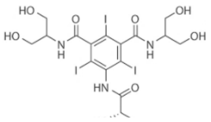	Neochema
28	**Iopromide**	73334-07-3	$C_{18}H_{24}I_3N_3O_8$ (791.1)	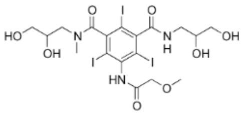	Neochema
29	**Ketoprofen**	22071-15-4	$C_{16}H_{14}O_3$ (254.3)	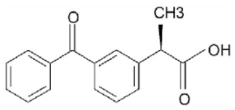	Neochema
30	**Lincomycin hydrochloride**	154-21-2	$C_{18}H_{34}N_2O_6S$ (406.5)	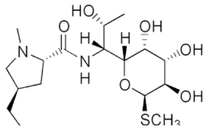	Neochema

	Analyte	CAS	Summary formula (Relative molecular	Structure	Supplier
31	**Loperamide hydrochloride**	34552-83-5	$C_{29}H_{33}ClN_2O_2 \cdot HCl$ (477.0)	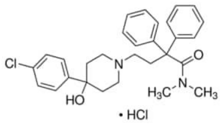	Chromservis
32	**Metoprolol tartrate**	37350-58-6	$C_{15}H_{25}NO_3 \cdot 1/2C_4H_6O_6$ (267.4)	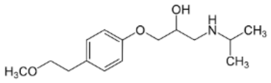	Neochema
33	**Metronidazole**	443-48-1	$C_6H_9N_3O_3$ (171.1)	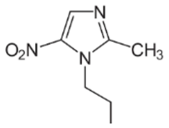	Neochema
34	**Mycophenolate Mofetil**	128794-94-5	$C_{23}H_{31}NO_7$ (433.5)	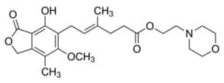	Merck
35	**Naproxen**	22204-53-1	$C_{14}H_{14}O_3$ (230.3)	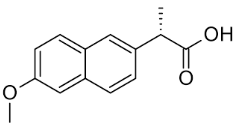	Neochema
36	**Oxazepam**	604-75-1	$C_{15}H_{11}ClN_2O_2$ (286.7)	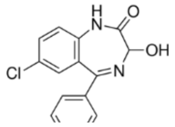	Chromservis
37	**Paclitaxel**	33069-62-4	$C_{47}H_{51}NO_{14}$ (853.9)	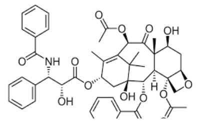	Chromservis
38	**Paracetamol (Acetaminophen)**	103-90-2	$C_8H_9NO_2$ (151.2)	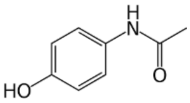	Neochema
39	**Piroxicam**	36322-90-4	$C_{15}H_{13}N_3O_4S$ (331.4)	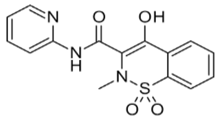	Neochema
40	**Propranolol hydrochloride**	525-66-6	$C_{16}H_{22}ClNO_2 \cdot HCl$ (259.3)	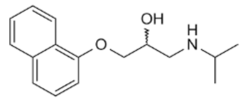	Neochema

	**Analyte**	**CAS**	**Summary formula (Relative molecular**	**Structure**	**Supplier**
41	**Salbutamol**	18559-94-9	$C_{13}H_{21}NO_3$ (239.3)	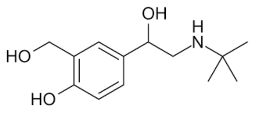	Neochema
42	**Sertraline hydrochloride**	79617-96-2	$C_{17}H_{17}Cl_2N \cdot HCl$ (306.2)	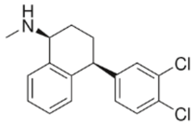	Merck
43	**Sotalol hydrochloride**	959-24-0	$C_{12}H_{20}N_2O_3S \cdot HCl$ (308.8)	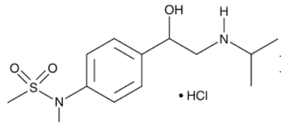	Neochema
44	**Sulfamethazine**	57-68-1	$C_{12}H_{14}N_4O_2S$ (278.3)	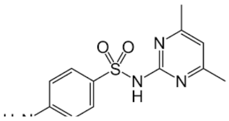	Neochema
45	**Sulfamethoxazole**	723-46-6	$C_{10}H_{11}N_3O_3S$ (253.3)	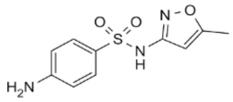	Neochema
46	**Terbutaline**	23031-25-6	$C_{12}H_{19}NO_3$ (225.3)	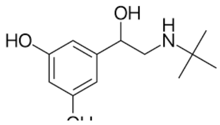	Neochema
47	**Thebaine**	115-37-7	$C_{19}H_{21}NO_3$ (311.4)	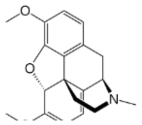	TEVA
48	**Tramadol hydrochloride**	36282-47-0	$C_{16}H_{25}NO_2 \cdot HCl$ (263.4)	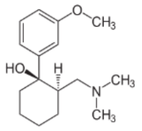	Neochema
49	**Trimethoprim**	738-70-5	$C_{14}H_{18}N_4O_3$ (290.3)	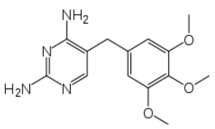	Neochema
50	**Valsartan**	137862-53-4	$C_{24}H_{29}N_5O_3$ (435.5)	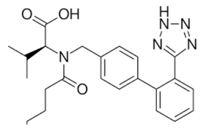	Neochema

	Analyte	CAS	Summary formula (Relative molecular	Structure	Supplier
51	**Warfarin**	81-81-2	$C_{19}H_{16}O_4$ (308.3)	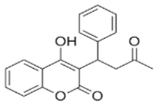	Neochema
52	**Zolpidem hemitartrate**	99294-93-6	$C_{19}H_{21}N_3O$ (307.4)	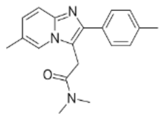	Chromservis

Table S2. List of ISTDs included in the validation and their characteristics - ISTD CAS number, summary formula, relative molecular weight and structure.

	Name	CAS	Summary formula (Relative molecular weight)	Structure	Supplier
1	**Atrazine D5**	163165-75-1	$C_8H_{14}ClN_5$ (220.7)	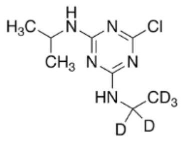	Chromservis
2	**Atrazine-desisopropyl D5**	1189961-78-1	$C_5H_8ClN_5$ (178.63)	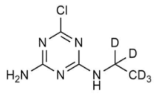	Chromservis
3	**Caffeine-13C**	78072-66-9	$^{13}C_3C_5H_{10}N_4O_2$ (197.17)	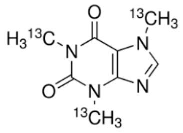	Neochema
4	**Carbamazepine D10**	132183-78-9	$C_{15}D_{10}H_2N_2O$ (246.33)	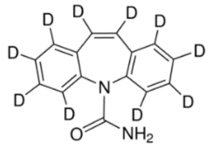	Neochema
5	**Carbendazim D4**	291765-95-2	$C_9H_9N_3O_2$ (195.21)	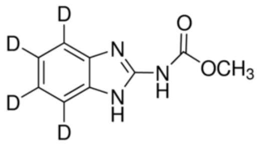	HPST

	Name	CAS	Summary formula (Relative molecular weight)	Structure	Supplier
6	**Citalopram D6 hydrobromide**	1190003-26-9	$C_{20}H_{15}D_6FN_2O \cdot HBr$ (330.43)	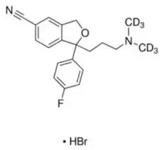	Chromservis
7	**Diclofenac D4**	153466-65-0	$C_{14}H_7D_4Cl_2NO_2$ (300.17)	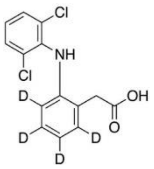	Neochema
8	**Methiocarb D3**	1581694-94-1	$C_{11}D_3H_{12}NO_2S$ (228.33)	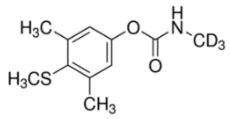	Merck
9	**Metolachlor D6**	1219803-97-0	$C_{15}H_{16}D_6ClNO_2$ (289.83)	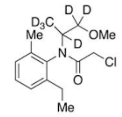	Chromservis
10	**Naproxen 13C D3**	1216704-11-8	$C_{13}{}^{13}CH_{11}D_3O_3$ (234.27)	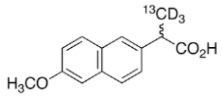	Chromservis
11	**Noreethindrone D6**	-	$C_{20}H_{20}D_6O_2$ (304.46)	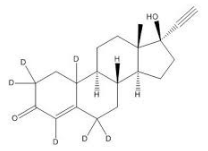	Chromservis
12	**Quizalofop-ethyl D3**	1398065-84-3	$C_{19}H_{14}D_3ClN_2O_4$ (375.82)	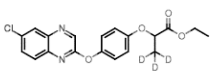	Chromservis
13	**Sulfamethoxazole D4**	1020719-86-1	$C_{10}H_7D_4N_3O_3S$ (257.30)	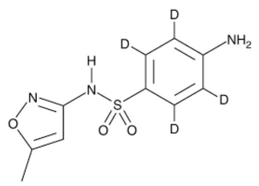	Neochema

	Name	CAS	Summary formula (Relative molecular weight)	Structure	Supplier
14	**Tebuconazole D6**	-	$C_{16}H_{16}ClN_3OD_6$ (313.86)	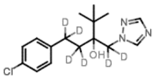	Chromservis

Table S3. Parameters of the MS/MS detection (MRM transitions of analytes, collision energy and retention time of analytes) - transitions marked in bold are used as quantification (* designation of precursor ions containing 2 isotopes of chlorine atoms).

Analyte	Ionization	Precursor ions (*m*/*z*)	Product ions (*m*/*z*)	Cone voltage (V)	Collision energy (V)	Retention time (t_R)
**Anastrozole**	ESI+	294.1	**225.0**/114.8	30	20/52	4.24
**Atenolol**	ESI+	267.0	**145.0**/190.0	30	23/16	2.55
**Azathioprine**	ESI+	278.0	**141.8**/232.0	30	11/13	2.92
**Bezafibrate**	ESI+	262.0	**139.0**/121.0	30	25/29	4.70
**Buprenorphine**	ESI+	468.3	**396.1**/414.2	30	39/32	3.83
**Butorphanol**	ESI+	328.0	**124.0**/282.0	30	26/29	3.47
**Caffeine**	ESI+	195.0	**138.0**/110.0	30	17/23	2.89
**Capecitabine**	ESI+	360.0	**173.8**/244.0	30	19/10	3.94
**Carbamazepine**	ESI+	237.0	**194.0**/179.0	35	20/35	4.16
**Citalopram**	ESI+	325.2	**109.0**/262.2	30	24/19	3.72
**Clofibric acid**	ESI-	213.0	**127.0**/85.0	30	15/11	4.59
**Cyclobenzaprine**	ESI+	276.0	**216.0**/231.0	30	24/16	3.93
**Cyclophosphamide**	ESI+	261.0	**140.0**/106.0	30	22/18	3.70
**Diazepam**	ESI+	285.0	**154.0**/193.0	30	26/30	4.94
**Diclofenac**	ESI+	294.0/296.0*	**250.0**/252.0	30	11/11	5.21

Analyte	Ionization	Precursor ions (*m*/*z*)	Product ions (*m*/*z*)	Cone voltage (V)	Collision energy (V)	Retention time (t_R)
**Enalapril**	ESI-	377.1	**234.0**/91.0	30	20/55	3.66
**Fluoxetine**	ESI+	310.1	**148.0**/44.0	30	8/7	4.05
**Flutamide**	ESI-	275.0	**202.0**/205.0	30	23/22	5.09
**Furosemide**	ESI-	329.0	**205.0**/285.0	30	22/14	4.06
**Gabapentin**	ESI+	172.2	**154.3**/137.3	25	15/18	2.72
**Gemfibrozil**	ESI-	249.0	**121.0**/127.0	30	15/10	5.47
**Hydrochlorothiazide**	ESI-	296.0	**269.0**/205.0	30	19/22	2.88
**Chloramphenicol**	ESI-	321.0	**152.1**/257.1	30	18/12	3.73
**Ifosfamide**	ESI+	261.0	**92.0**/154.0	30	25/21	3.64
**Indomethacin**	ESI+	358.0	**139.0**/174.0	30	22/11	5.20
**Iomeprol**	ESI+	777.8	**405.0**/531.8	30	38/30	2.46
**Iopamidol**	ESI+	777.8	**387.0**/313.8	30	39/52	1.93
**Iopromide**	ESI+	791.8	**300.0**/572.8	30	56/23	2.65
**Ketoprofen**	ESI+	255.1	**209.0**/105.0	30	15/23	4.67
**Lincomycin**	ESI+	407.0	**126.0**/359.0	35	20/18	2.83
**Loperamide**	ESI+	477.2	**210.1**/266.1	30	45/12	4.29
**Metoprolol**	ESI+	268.2	**116.0**/72.0	30	18/22	3.25
**Metronidazole**	ESI+	172.0	**128.0**/82.0	30	15/21	2.71
**Mycophenolate Mofetil**	ESI+	434.0	**114.0**/195.0	30	24/33	3.82
**Naproxen**	ESI+	231.0	**185.0**/170.0	30	14/29	4.63
**Oxazepam**	ESI+	287.0	**241.0**/269.0	30	23/15	4.30
**Paclitaxel**	ESI+	876.4	**308.1**/591.3	30	27/23	5.08

**Analyte**	**Ionization**	**Precursor ions (*m*/*z*)**	**Product ions (*m*/*z*)**	**Cone voltage (V)**	**Collision energy (V)**	**Retention time (t_R)**
**Paracetamol (Acetaminophen)**	ESI+	152.0	**110.0**/65.0	30	15/30	2.68
**Piroxicam**	ESI+	332.0	**95.0**/121.0	30	18/25	4.33
**Propranolol**	ESI+	260.0	**116.0**/183.0	30	16/16	3.65
**Salbutamol**	ESI+	240.0	**148.0**/166.0	30	20/12	2.52
**Sertraline**	ESI+	306.0	**159.0**/275.0	30	23/11	4.04
**Sotalol**	ESI+	273.0	**133.0**/213.0	30	27/17	2.52
**Sulfamethazine**	ESI+	279.0	**124.0**/186.0	30	20/18	3.26
**Sulfamethoxazole**	ESI+	254.0	**156.0**/92.0	30	16/26	3.58
**Terbutaline**	ESI+	226.1	**152.0**/107.0	30	16/26	2.47
**Thebaine**	ESI+	312.0	**58.0**/266.0	30	30/40	3.29
**Tramadol**	ESI+	264.0	**58.0**/246.0	30	15/10	3.26
**Trimethoprim**	ESI+	291.0	**230.0**/123.0	39	24/27	2.90
**Valsartan**	ESI+	436.0	**207.0**/235.0	30	28/22	4.72
**Warfarin**	ESI+	309.0	**163.0**/251.0	30	14/22	4.91
**Zolpidem**	ESI+	308.0	**235.0**/263.0	30	33/27	3.44
**Atrazine D5**	ESI+	221.0/223.0*	**179.0**/181.0	35	18/18	4.53
**Atrazine-desisopropyl D5**	ESI+	179.0	**101.0**/137.0	35	18/16	3.10
**Caffeine-13C**	ESI+	198.0	**140.0**/112.0	30	19/22	2.89
**Carbamazepine D10**	ESI+	247.0	**204.0**/201.0	30	21/22	4.14
**Carbendazim D4**	ESI+	196.0	**164.0**/136.0	35	20/28	2.90
**Citalopram D6**	ESI+	331.3	**109.1**/262.2	30	26/19	3.78
**Diclofenac D4**	ESI-	298.0/300.0*	**254.0**/256.0	30	12/12	5.20

**Analyte**	**Ionization**	**Precursor ions (*m*/*z*)**	**Product ions (*m*/*z*)**	**Cone voltage (V)**	**Collision energy (V)**	**Retention time (t_R)**
**Phosalon D10**	ESI+	378.0	**182.0**/111.0	35	16/38	5.77
**Methiocarb D3**	ESI+	229.0	**169.0**/121.0	30	19/17	4.96
**Metolachlor D6**	ESI+	290.0	**258.0**/182.0	35	16/25	5.39
**Naproxen 13C D3**	ESI+	235.0	**189.0**/170.0	30	15/25	4.62
**Noreethindrone D6**	ESI+	305.3	**113.0**/237.2	30	30/21	4.71
**Quizalofop D3**	ESI+	348.0	**302.0**/244.0	35	18/31	5.07
**Sulfamethoxazole D4**	ESI+	258.0	**160.0**/96.0	30	16/26	3.57
**Tebuconazole D6**	ESI+	314.0	**71.8**/124.8	30	21/38	5.24

Table S4. Final operating parameters of UHPLC-MS/MS method.

**MS conditions**	Heat block temperature		150 °C
	Column voltage		0.75 kV
	Desolvation temperature		600 °C
	Desolvation gas flow		800 L hod^{-1}
	Cone gas flow		150 L hod^{-1}
	Nebulizer gas		7.0 bar
	Collision gas flow		0.19 L min^{-1}
	MRM window		0.4 min
**LC conditions**	Column		Acquity UPLC BEH C18 (2.1 mm × 100 mm × 1.7 μm)
	Column temperature		40 °C
	Injection volume		50 μL
	Flow rate		0.4 mL min^{-1}
	Total run time		9 min
	Mobile phase	A	0.01% formic acid in Milli-Q water

	B	methanol						
Gradient profile	Time (min)	0	0.5	5.0	5.1	7.0	7.1	9.0
	A (%)	98	98	5	0	0	98	98
	B (%)	2	2	95	100	100	2	2

Table S5. List of ISTDs and assigned analytes in wastewater matrices.

**ISTD – wastewater**	**Analyte**
Atrazine D5	Anastrozole
	Bezafibrate
	Capecitabine
	Cyclophosphamide
	Diazepam
	Enalapril
	Indomethacin
	Oxazepam
	Piroxicam
	Sulfamethazine
Atrazine-desisopropyl D5	Iopamidol
	Paracetamol (Acetaminophen)
Caffeine-13C	Caffeine
	Ketoprofen
Carbamazepine D10	Buprenorphine
	Carbamazepine
	Cyklobenzaprine
	Fluoxetine
	Iopromide
	Valsartan
Carbendazim D4	Flutamide
	Gabapentin
	Hydrochlorothiazid
	Chloramphenicol
	Thebaine
Citalopram D6	Atenolol

**ISTD – wastewater**	**Analyte**
	Azathioprine
	Butorphanol
	Citalopram
	Clofibric acid
	Diclofenac
	Furosemide
	Iomeprol
	Loperamide
	Salbutamol
	Sotalol
	Terbutaline
Diclofenac D4	Tramadol
	Trimethoprim
	Zolpidem
Methiocarb D3	Metronidazole
Metolachlor D6	Gemfibrozil
Naproxen 13C D3	Naproxen
Noreethindrone D6	Paclitaxel
	Propranolol
	Sertraline
Quizalofop D3	Metoprolol
Sulfamethoxazole D4	Fluoxetine
	Sulfamethoxazole
Tebuconazole D6	Ifosfamide
	Lincomycin
	Mycophenolate Mofetil
	Warfarin

Table S6. Overview of values of limits of detection and determination for individual analytes in wastewater.

Analyte	Average [ng L^{-1}]	SD [ng L^{-1}]	LOD [ng L^{-1}]	LOQ [ng L^{-1}]
**Anastrozole**	8.52	0.12	0.456	1.37
**Atenolol**	5.03	0.61	2.29	6.85
**Azathioprine**	7.37	0.53	1.99	5.98
**Bezafibrate**	8.14	0.27	0.99	2.98
**Buprenorphine**	11.86	1.00	3.75	11.25
**Butorphanol**	6.68	0.36	1.34	4.03
**Caffeine**	744.37	22.31	83.59	250.77
**Capecitabine**	153.59	10.92	40.92	122.77
**Carbamazepine**	23.27	0.21	0.78	2.33
**Citalopram**	7.42	0.10	0.38	1.14
**Clofibric acid**	26.71	5.33	19.96	59.87
**Cyclobenzaprine**	99.33	4.32	16.19	48.56
**Cyclophosphamide**	10.17	0.62	2.31	6.92
**Diazepam**	9.81	0.11	0.42	1.27
**Diclofenac**	20.18	3.67	13.77	41.30
**Enalapril**	8.80	0.38	1.41	4.22
**Fluoxetine**	80.93	2.94	11.00	33.00
**Flutamide**	4.43	0.20	0.76	2.29
**Furosemide**	34.06	4.66	17.45	52.36
**Gabapentin**	432.50	4.73	17.72	53.15
**Gemfibrozil**	2.65	0.53	1.98	5.94
**Hydrochlorothiazide**	46.21	3.64	13.62	40.86
**Chloramfenicol**	4.45	0.51	1.91	5.73
**Ifosfamide**	9.98	0.59	2.20	6.60
**Indomethacin**	8.03	0.38	1.44	4.31
**Iomeprol**	548.52	5.66	21.21	63.64

**Iopamidol**	9.41	1.61	6.04	18.13
**Iopromide**	28.66	5.20	19.49	58.47
**Ketoprofen**	29.85	1.14	4.26	12.77
**Lincomycin**	7.63	0.25	0.94	2.81
**Loperamide**	128.29	1.84	6.88	20.63
**Metoprolol**	11.30	0.35	1.30	3.91
**Metronidazole**	3.40	0.21	0.80	2.38
**Mycophenolate Mofetil**	9.69	1.18	4.42	13.24
**Naproxen**	67.95	9.02	33.79	101.36
**Oxazepam**	8.38	0.28	1.04	3.11
**Paclitaxel**	66.95	13.03	48.81	146.43
**Paracetamol**	19.97	0.88	3.30	9.90
**Piroxicam**	8.56	0.13	0.48	1.45
**Propranolol**	8.20	0.36	1.33	4.00
**Salbutamol**	8.07	0.27	1.00	3.00
**Sertraline**	66.28	4.45	16.69	50.06
**Sotalol**	18.45	0.28	1.04	3.10
**Sulfamethazine**	9.34	0.32	1.21	3.62
**Sulfamethoxazole**	10.99	0.47	1.75	5.24
**Terbutaline**	2.51	0.18	0.66	1.97
**Thebaine**	6.49	0.63	2.37	7.12
**Tramadol**	18.52	0.46	1.71	5.14
**Trimethoprim**	6.81	0.29	1.08	3.25
**Valsartan**	21.36	1.37	5.15	15.44
**Warfarin**	9.67	0.15	0.58	1.73
**Zolpidem**	7.13	0.12	0.45	1.35

Table S7. Overview of linear range, coefficient of determination (R2) values and working range for individual analytes in wastewater.

**Analyte**	**Linear range [ng L^{-1}]**	**Coefficient of determination (R^2)**	**Working range for wastewaters [ng L^{-1}]**
**Anastrozole**	2.5 – 500	0.9998	2.5 – 500
**Atenolol**	2.5 – 500	0.9997	10.0 – 500
**Azathioprine**	2.5 – 500	0.9997	10.0 – 500
**Bezafibrate**	2.5 – 1000	0.9999	5.0 – 1000
**Buprenorphine**	2.5 – 1000	0.9994	25.0 – 1000
**Butorphanol**	2.5 – 1000	0.9997	5.0 – 1000
**Caffeine**	50.0 – 1000	0.9988	250.0 – 1000
**Capecitabine**	5.0 – 1000	0.9992	250.0 – 1000
**Carbamazepine**	2.5 – 1000	1.0000	2.5 – 1000
**Citalopram**	2.5 – 1000	0.9993	2.5 – 1000
**Clofibric acid**	50.0 – 1000	0.9996	75.0 – 1000
**Cyclobenzaprine**	50.0 – 1000	0.9997	50.0 – 1000
**Cyclophosphamide**	2.5 – 1000	0.9999	10.0 – 1000
**Diazepam**	2.5 – 1000	0.9999	2.5 – 1000
**Diclofenac**	5.0 – 1000	0.9996	75.0 – 1000
**Enalapril**	2.5 – 1000	0.9999	5.0 – 1000
**Fluoxetine**	50.0 – 1000	0.9993	50.0 – 1000
**Flutamide**	2.5 – 1000	0.9999	2.5 – 1000
**Furosemide**	5.0 – 1000	0.9995	75.0 – 1000
**Gabapentin**	50.0 – 1000	0.9997	75.0 – 1000
**Gemfibrozil**	5.0 – 1000	0.9997	10.0 – 1000
**Hydrochlorothiazide**	2.5 – 500	0.9997	50.0 – 500
**Chloramfenikol**	2.5 – 1000	0.9992	10.0 – 1000
**Ifosfamide**	2.5 – 1000	0.9999	10.0 – 1000
**Indomethacin**	2.5 – 1000	0.9999	5.0 – 500
**Iomeprol**	50.0 – 1000	0.9994	75.0 – 1000

**Analyte**	**Linear range [ng L⁻¹]**	**Coefficient of determination (R^2)**	**Working range for wastewaters [ng L⁻¹]**
**Iopamidol**	2.5 – 1000	0.9991	25.0 – 1000
**Iopromide**	2.5 – 500	0.9991	75.0 – 500
**Ketoprofen**	2.5 – 1000	0.9998	25.0 – 1000
**Lincomycin**	2.5 – 1000	0.9999	5.0 – 1000
**Loperamide**	50.0 – 1000	0.9997	25.0 – 1000
**Metoprolol**	2.5 – 1000	0.9996	5.0 – 1000
**Metronidazole**	2.5 – 500	0.9999	2.5 – 500
**Mycophenolate Mofetil**	2.5 – 1000	0.9998	25.0 – 1000
**Naproxen**	50.0 – 1000	0.9993	100.0 – 1000
**Oxazepam**	2.5 – 1000	0.9999	5.0 – 1000
**Paclitaxel**	5.0 – 1000	0.9995	250.0 – 1000
**Paracetamol**	2.5 – 500	0.9998	10.0 – 500
**Piroxicam**	2.5 – 1000	1.0000	2.5 – 1000
**Propranolol**	2.5 – 1000	0.9998	5.0 – 1000
**Salbutamol**	2.5 – 500	0.9997	5.0 – 500
**Sertraline**	50.0 – 1000	0.9990	75.0 – 1000
**Sotalol**	2.5 – 500	0.9996	5.0 – 500
**Sulfamethazine**	2.5 – 1000	0.9999	5.0 – 1000
**Sulfamethoxazole**	2.5 – 1000	0.9999	10.0 – 1000
**Terbutaline**	2.5 – 500	0.9997	2.5 – 500
**Thebaine**	2.5 – 1000	0.9998	10.0 – 1000
**Tramadol**	2.5 – 1000	0.9998	10.0 – 1000
**Trimethoprim**	2.5 – 1000	0.9999	5.0 – 1000
**Valsartan**	2.5 – 1000	0.9999	25.0 – 1000
**Warfarin**	2.5 – 1000	0.9999	2.5 – 1000
**Zolpidem**	2.5 – 500	0.9998	2.5 – 500

Table S8. Summary of repeatability results expressed as RSD (%). average of measured concentrations of a given analyte at one concentration level and standard deviation of individual analytes in MQ water.

Analyte	MQ water					
	L1 (0.01 ng mL^{-1})			L2 (0.1 ng mL^{-1})		
	Average [ng L^{-1}]	SD [ng L^{-1}]	RSD [%]	Average [ng L^{-1}]	SD [ng L^{-1}]	RSD [%]
**Anastrozole**	9.57	0.26	2.69	94.09	2.56	2.72
**Atenolol**	11.47	0.88	7.67	113.00	4.05	3.58
**Azathioprine**	9.87	0.48	4.86	94.84	2.02	2.13
**Bezafibrate**	8.88	0.86	9.67	82.09	6.82	8.31
**Buprenorphine**	12.04	0.40	3.34	113.34	6.63	5.85
**Butorphanol**	11.92	1.12	9.42	115.00	7.19	6.25
**Caffeine**	n.d.*	-	-	95.94	15.60	17.15
**Capecitabine**	n.d.*	-	-	88.04	17.02	19.34
**Carbamazepine**	9.54	0.36	3.80	95.47	1.75	1.83
**Citalopram**	13.39	1.37	10.23	112.12	5.22	4.66
**Clofibric acid**	n.d.*	-	-	66.05	12.59	19.07
**Cyclobenzaprine**	n.d.*	-	-	67.31	7.70	11.44
**Cyclophosphamide**	9.26	0.56	6.05	95.57	2.51	2.63
**Diazepam**	9.79	0.19	1.96	93.73	1.90	2.03
**Diclofenac**	n.d.*	-	-	77.64	10.55	13.59
**Enalapril**	9.89	0.46	4.62	98.87	1.53	1.55
**Fluoxetine**	n.d.*	-	-	93.57	17.08	18.26
**Flutamide**	8.69	0.55	6.31	80.21	9.53	11.88
**Furosemide**	8.14	1.47	18.00	68.79	11.83	17.20
**Gabapentin**	n.d.*	-	-	88.59	2.97	3.36
**Gemfibrozil**	10.59	1.05	9.88	90.92	5.25	5.77
**Hydrochlorothiazide**	n.d.*	-	-	97.86	6.27	6.41
**Chloramfenikol**	11.42	1.39	12.14	92.78	4.56	4.92
**Ifosfamide**	10.02	0.62	6.16	95.11	2.32	2.44
**Indomethacin**	8.44	0.70	8.28	73.56	8.58	11.66
**Iomeprol**	n.d.*	-	-	87.95	25.87	16.38
**Iopamidol**	9.96	0.92	9.26	103.09	0.92	0.89
**Iopromide**	8.13	1.09	13.46	105.72	5.11	4.83
**Ketoprofen**	7.79	0.58	7.48	76.60	7.72	10.07
**Lincomycin**	10.28	0.64	6.24	100.96	1.68	1.67
**Loperamide**	n.d.*	-	-	145.68	19.76	13.56
**Metoprolol**	11.55	0.90	7.77	103.46	2.72	2.63
**Metronidazole**	9.90	0.19	1.88	98.19	0.74	0.75
**Mycophenolate Mofetil**	8.10	1.50	18.52	86.08	4.92	5.72
**Naproxen**	n.d.*	-	-	79.33	6.37	8.03
**Oxazepam**	9.56	0.32	3.30	94.31	2.40	2.54
**Paclitaxel**	n.d.*	-	-	74.46	12.44	19.29

Analyte	MQ water					
	L1 (0.01 ng mL^{-1})			L2 (0.1 ng mL^{-1})		
	Average [ng L^{-1}]	SD [ng L^{-1}]	RSD [%]	Average [ng L^{-1}]	SD [ng L^{-1}]	RSD [%]
**Paracetamol**	9.25	1.20	12.96	92.01	1.32	1.43
**Piroxicam**	9.93	0.31	3.09	97.59	1.10	1.12
**Propranolol**	9.42	0.63	6.65	82.26	10.54	16.94
**Salbutamol**	11.96	0.93	7.81	113.13	3.82	3.38
**Sertraline**	n.d.*	-	-	73.65	14.35	19.49
**Sotalol**	10.97	0.81	7.37	107.08	3.48	3.25
**Sulfamethazine**	9.68	0.24	2.49	97.16	1.85	1.91
**Sulfamethoxazole**	9.46	0.55	5.78	97.58	1.44	1.48
**Terbutaline**	11.93	1.37	11.46	117.22	6.27	5.35
**Thebaine**	11.92	1.38	11.61	106.99	5.53	5.17
**Tramadol**	11.54	1.09	9.44	105.59	3.67	3.48
**Trimethoprim**	8.04	1.19	14.81	77.05	8.88	11.52
**Valsartan**	9.09	1.27	14.00	82.81	7.58	9.15
**Warfarin**	8.04	0.51	6.36	80.45	3.35	4.16
**Zolpidem**	9.29	0.68	7.32	88.92	4.02	4.52

n.d. - detection limit of the analyte is higher than the lower concentration level of the spike L1 = 0.01 μg L^{-1}*

Table S9. Summary of repeatability results expressed as RSD (%), average of measured concentrations of a given analyte at one concentration level and standard deviation of individual analytes in wastewater.

Analyte	Wastewater					
	L1 (0.01 ng mL^{-1})			L2 (0.1 ng mL^{-1})		
	Average [ng L^{-1}]	SD [ng L^{-1}]	RSD [%]	Average [ng L^{-1}]	SD [ng L^{-1}]	RSD [%]
**Anastrozole**	8.52	0.12	1.43	81.00	1.91	2.36
**Atenolol**	5.03	0.61	12.11	30.53	0.97	3.16
**Azathioprine**	7.37	0.53	7.21	64.64	2.35	3.64
**Bezafibrate**	8.14	0.26	3.25	71.90	5.85	8.13
**Buprenorphine**	11.86	1.00	8.44	99.54	3.28	3.29
**Butorphanol**	6.68	0.36	5.37	61.04	1.11	1.81
**Caffeine**	n.d.*	-	-	744.37*	22.31	3.00
**Capecitabine**	n.d.*	-	-	153.59	10.92	7.11
**Carbamazepine**	23.27	0.21	0.89	110.08	1.54	1.40
**Citalopram**	7.42	0.10	1.37	66.50	2.97	4.47
**Clofibric acid**	n.d.*	-	-	6.71	5.33	19.94
**Cyclobenzaprine**	n.d.*	-	-	99.33	4.32	4.35
**Cyclophosphamide**	10.17	0.62	6.05	94.52	2.41	2.55
**Diazepam**	9.81	0.11	1.15	92.16	2.10	2.28

**Analyte**	**Wastewater**					
	**L1 (0.01 ng mL^{-1})**			**L2 (0.1 ng mL^{-1})**		
	**Average [ng L^{-1}]**	**SD [ng L^{-1}]**	**RSD [%]**	**Average [ng L^{-1}]**	**SD [ng L^{-1}]**	**RSD [%]**
**Diclofenac**	n.d.*	-	-	20.18	3.67	18.20
**Enalapril**	8.80	0.38	4.27	88.00	1.98	2.25
**Fluoxetine**	n.d.*	-	-	80.93	2.94	3.63
**Flutamide**	4.43	0.20	4.61	37.89	5.96	15.72
**Furosemide**	n.d.*	-	-	34.06	4.66	13.68
**Gabapentin**	n.d.*	-	-	475.92*	11.80	2.48
**Gemfibrozil**	n.d.*	-	-	2.65	0.53	19.95
**Hydrochlorothiazide**	n.d.*	-	-	46.21	3.63	7.87
**Chloramfenikol**	4.45	0.51	11.46	26.17	1.79	6.86
**Ifosfamide**	9.98	0.59	5.89	103.61	3.16	3.05
**Indomethacin**	8.03	0.38	4.78	73.28	7.34	10.02
**Iomeprol**	n.d.*	-	-	596.92*	13.94	2.33
**Iopamidol**	9.41	1.61	17.13	48.60	1.12	2.30
**Iopromide**	28.66	5.20	18.15	105.09	7.07	6.72
**Ketoprofen**	29.85	1.14	3.81	89.62	7.42	8.28
**Lincomycin**	7.63	0.25	3.28	89.42	1.65	1.84
**Loperamide**	n.d.*	-	-	128.29	1.83	1.43
**Metoprolol**	11.30	0.35	3.08	66.77	2.84	4.26
**Metronidazole**	3.40	0.21	6.25	48.83	1.16	2.37
**Mycophenolate Mofetil**	9.69	1.18	12.16	92.40	4.87	5.27
**Naproxen**	n.d.*	-	-	67.95	9.02	13.27
**Oxazepam**	8.38	0.28	3.30	83.56	2.66	3.18
**Paclitaxel**	n.d.*	-	-	66.95	13.03	19.46
**Paracetamol**	19.97	0.88	4.41	69.11	0.90	1.31
**Piroxicam**	8.56	0.13	1.51	82.81	1.53	1.84
**Propranolol**	8.20	0.36	4.34	74.95	1.47	1.96
**Salbutamol**	n.d.*	-	-	8.07	0.27	3.30
**Sertraline**	n.d.*	-	-	66.28	4.45	6.72
**Sotalol**	n.d.*	-	-	18.45	0.28	1.50
**Sulfamethazine**	9.34	0.32	3.45	85.05	1.89	2.22
**Sulfamethoxazole**	10.99	0.47	4.24	82.00	2.92	3.56
**Terbutaline**	n.d.*	-	-	2.51	0.17	6.97
**Thebaine**	6.49	0.63	9.76	50.56	3.76	7.44
**Tramadol**	18.52	0.46	2.47	62.48	1.07	1.71
**Trimethoprim**	6.81	0.29	4.24	62.59	0.71	1.13
**Valsartan**	21.36	1.37	6.43	92.11	8.21	8.91
**Warfarin**	9.67	0.15	1.59	85.04	5.99	7.05
**Zolpidem**	7.13	0.12	1.68	68.20	1.62	2.38

n.d. - detection limit of the analyte is higher than the lower concentration level of the spike L1 = 0.01 μg L^{-1}*

** The given analytes were determined in raw validation samples at levels that were many times higher than the level of fortification. For these analytes. RSD (%) values were calculated from concentrations determined by repeated spike analysis.*

Table S10. Method accuracy results expressed as recovery for MQ and wastewater.

Analyte	Recovery [%]			
	MQ water		Wastewater	
	L1	L2	L1	L2
**Anastrozole**	100.1	102.4	97.0	98.7
**Atenolol**	84.0	96.7	64.6	45.8
**Azathioprine**	104.9	103.1	94.5	97.1
**Bezafibrate**	92.9	89.4	92.8	87.6
**Buprenorphine**	88.1	97.0	103.0	98.2
**Butorphanol**	87.2	98.4	85.7	91.7
**Caffeine**	n.d.*	91.7	n.d.**	n.d.**
**Capecitabine**	n.d.*	95.8	n.d.*	187.1
**Carbamazepine**	97.7	99.5	202.1	108.6
**Citalopram**	98.0	95.9	95.3	99.9
**Clofibric acid**	n.d.*	84.9	n.d.*	66.0
**Cyclobenzaprine**	n.d.*	73.1	n.d.*	98.0
**Cyclophosphamide**	96.9	104.0	103.4	105.6
**Diazepam**	102.4	102.0	99.8	103.0
**Diclofenac**	n.d.*	97.0	n.d.*	30.3
**Enalapril**	103.5	107.6	100.3	107.2
**Fluoxetine**	n.d.*	97.5	n.d.*	100.0
**Flutamide**	91.0	87.3	71.7	62.8
**Furosemide**	90.2	81.6	n.d.*	84.1
**Gabapentin**	n.d.*	96.3	n.d.**	n.d.**
**Gemfibrozil**	108.9	97.3	n.d.*	3.6
**Hydrochlorothiazide**	n.d.*	104.9	n.d.*	76.5

**Analyte**	**Recovery [%]**			
	**MQ water**		**Wastewater**	
	**L1**	**L2**	**L1**	**L2**
**Chloramfenikol**	121.3	100.8	72.1	43.4
**Ifosfamide**	106.5	103.4	101.5	115.8
**Indomethacin**	88.3	80.1	91.5	89.3
**Iomeprol**	n.d.*	135.1	n.d.**	n.d.**
**Iopamidol**	103.4	104.0	106.9	66.4
**Iopromide**	84.4	106.6	249.0	103.6
**Ketoprofen**	86.3	90.8	246.9	107.1
**Lincomycin**	105.2	105.3	77.6	99.9
**Loperamide**	n.d.*	124.6	n.d.*	192.7
**Metoprolol**	84.5	88.5	105.2	83.0
**Metronidazole**	105.2	106.7	88.5	74.5
**Mycophenolate Mofetil**	83.3	88.7	98.5	103.2
**Naproxen**	n.d.*	98.4	n.d.*	92.1
**Oxazepam**	100.0	102.7	95.4	101.8
**Paclitaxel**	n.d.*	82.9	n.d.*	85.6
**Paracetamol**	94.6	95.9	226.7	94.4
**Piroxicam**	103.9	106.2	97.6	100.9
**Propranolol**	14.7	80.1	82.2	95.8
**Salbutamol**	87.5	96.8	n.d.*	12.1
**Sertraline**	n.d.*	94.7	n.d.*	95.4
**Sotalol**	80.3	91.6	n.d.*	27.7
**Sulfamethazine**	101.3	105.8	106.4	103.6
**Sulfamethoxazole**	97.2	100.6	105.7	101.4
**Terbutaline**	87.3	100.3	n.d.*	3.8
**Thebaine**	87.2	91.5	105.2	83.8
**Tramadol**	84.5	90.3	237.6	93.8

**Analyte**	**Recovery [%]**			
	**MQ water**		**Wastewater**	
	**L1**	**L2**	**L1**	**L2**
**Trimethoprim**	85.5	83.7	87.4	94.0
**Valsartan**	93.1	86.3	185.5	90.8
**Warfarin**	81.7	86.3	98.4	95.0
**Zolpidem**	98.7	96.6	91.5	102.4

n.d. - detection limit of the analyte is higher than the lower concentration level of the spike L1 = 0.01 µg L^{-1}*

*n.d.** - For the mentioned analytes, it was not possible to determine the exact recovery value due to the fact that the samples used for validation were contaminated with these substances at quite high levels, far exceeding the concentration at which the samples were fortified. Therefore, the recoveries of these analytes can only be determined from the analysis of MQ water.*
